# THE DYNAMIC GENOME-CHALLENGES AND OPPORTUNITIES FOR HEALTHY AGING

**DOI:** 10.1093/geroni/igac059.104

**Published:** 2022-12-20

**Authors:** Peter Adams

**Affiliations:** Sanford Burnham, San Diego, California, United States

## Abstract

Chromatin is a key determinant of cell phenotype and function. Therefore, chromatin stability over the lifespan is presumably a pre-requisite for maintenance of cell and tissue phenotype and function, and hence healthy aging and longevity. However, chromatin is not a static fixed structure, but is a dynamic and plastic “breathing” assembly. This dynamic chromatin likely represents a challenge for a cell to achieve phenotypic stability, healthy aging and longevity. As a dynamic and plastic entity, chromatin is prone to change or drift, a process likely exacerbated by intrinsic cellular processes and extrinsic/environmental influences. We have proposed that cells possess mechanisms of chromatin homeostasis, or chromostasis, that preserve chromatin integrity, suppress phenotypic instability and so slow the pace of aging. Implications of dynamic chromatin for healthy aging and longevity will be discussed.

